# Predicting Key Agronomic Soil Properties with UV-Vis Fluorescence Measurements Combined with Vis-NIR-SWIR Reflectance Spectroscopy: A Farm-Scale Study in a Mediterranean Viticultural Agroecosystem

**DOI:** 10.3390/s18041157

**Published:** 2018-04-10

**Authors:** Emmanuelle Vaudour, Zoran G. Cerovic, Dav M. Ebengo, Gwendal Latouche

**Affiliations:** 1 UMR ECOSYS, AgroParisTech, INRA, Université Paris-Saclay, 78850 Thiverval-Grignon, France; dav.ebengomwampongo@agroparistech.fr; 2 Ecologie, Systématique et Evolution (UMR 8079), CNRS, Univ. Paris-Sud, AgroParisTech, Université Paris-Saclay, 91400 Orsay, France; zoran.cerovic@u-psud.fr (Z.G.C.); gwendal.latouche@u-psud.fr (G.L.)

**Keywords:** UV-Vis fluorescence, multiple excitation fluorescence sensor, Vis-NIR-SWIR reflectance spectroscopy, soil properties, partial least squares regression, Mediterranean vineyard soils, fertility assessment, model averaging

## Abstract

For adequate crop and soil management, rapid and accurate techniques for monitoring soil properties are particularly important when a farmer starts up his activities and needs a diagnosis of his cultivated fields. This study aimed to evaluate the potential of fluorescence measured directly on 146 whole soil solid samples, for predicting key soil properties at the scale of a 6 ha Mediterranean wine estate with contrasting soils. UV-Vis fluorescence measurements were carried out in conjunction with reflectance measurements in the Vis-NIR-SWIR range. Combining PLSR predictions from Vis-NIR-SWIR reflectance spectra and from a set of fluorescence signals enabled us to improve the power of prediction of a number of key agronomic soil properties including SOC, N_tot_, CaCO_3_, iron, fine particle-sizes (clay, fine silt, fine sand), CEC, pH and exchangeable Ca^2+^ with cross-validation RPD ≥ 2 and *R²* ≥ 0.75, while exchangeable K^+^, Na^+^, Mg^2+^, coarse silt and coarse sand contents were fairly predicted (1.42 ≤ RPD < 2 and 0.54 ≤ *R²* < 0.75). Predictions of SOC, N_tot_, CaCO_3_, iron contents, and pH were still good (RPD ≥ 1.8, *R²* ≥ 0.68) when using a single fluorescence signal or index such as SFR_R or FERARI, highlighting the unexpected importance of red excitations and indices derived from plant studies. The predictive ability of single fluorescence indices or original signals was very significant for topsoil: this is very important for a farmer who wishes to update information on soil nutrient for the purpose of fertility diagnosis and particularly nitrogen fertilization. These results open encouraging perspectives for using miniaturized fluorescence devices enabling red excitation coupled with red or far-red fluorescence emissions directly in the field.

## 1. Introduction

In order to enable adequate crop and soil management, rapid, accurate techniques are needed for the quantification and monitoring of soil properties. This is particularly important when a farmer starts up his activities and needs a diagnosis of the soil properties that characterize the cultivated fields. Soil properties may be accurately predicted from laboratory reflectance spectroscopy in the visible (Vis, 400–700 nm), near-infrared (NIR, 700–1100 nm), and short-wave infrared (SWIR, 1100–2500 nm) ranges [1,2,3]. Fluorescence spectroscopy may be either an alternative to or complementary to reflectance spectroscopy. As a matter of fact, fluorescence spectroscopy consists of measuring the photoluminescence of molecules that emit light after having absorbed ultraviolet, visible, or infrared light [4,5,6]. The emission spectrum is expected to overlap with the absorption spectrum at a wavelength corresponding to the lowest vibrational transition level and the rest of the emission spectrum is expected to be of lower energy, or longer wavelength. Among soil components, organic matter components such as humic and fulvic acids have fluorescent properties [7,8]. Such fluorescent behavior relies on the aromaticity, aliphatic character, degree of polycondensation, content of carboxylic groups or organic free radicals, or presence of amide groups or polysaccharidic structures [9,10,11]. In particular, emission spectra shift towards longer wavelengths with increasing humification [11,12,13]. Most studies involving fluorescence spectroscopy of soils have been carried out on liquid extracts of soil samples and were devoted to soil organic compounds [11,14,15,16]. Few studies relied on fluorescence measurements performed on solid soil samples or “whole soil samples,” with the exception of Van Vliet-Lanöe [17], for the purpose of applying fluorescence observations to soil micromorphology, or McMurtrey et al. [18] and Daughtry et al. [19], for the purpose of discriminating crop residues from soils. McMurtrey et al. [18] and Daughtry et al. [19] illuminated solid samples of soil and crop residues placed in dishes with a 20-cm diameter with ultraviolet radiation: as a matter of fact, crop residues younger than two years old contain lignin and riboflavin, and fluoresce more than soils, specifically in the blue-green range centered between 420 nm to 520 nm induced by excitation wavelengths centered between 350 nm to 400 nm [19]. Using laser-induced fluorescence (LIF), Milori et al. [13] also excited topsoil solid samples of Brazilian oxisols with 351-nm ultraviolet radiation and recorded emitted signals in the visible region. They found a correlation between C content and the area of the LIF spectra (*R²* = 0.66). Until now however, fluorescence spectroscopy has seldom been used for detecting soil compounds other than fresh or humified organic matter. Yet, some minerals present in soils, such as calcite, gypsum, halite, and quartz, also have fluorescent properties [20,21]; in particular, clay coatings associated with weakly evolved polymers of aluminum will strongly fluoresce in blue and UV lights [17]. Moreover, secondary calcite in or around root channels might be related to organic inclusions [17,22]. Using liquid extracts, Brunetti et al. [16] isolated humic acids from clay-size and silt-size fractions of fersiallitic soils and found that the presence of highly polycondensated humic acids macromolecules less susceptible to degradation occurred in the silt-size fraction. Because humified organic matter is tightly bound to clay or either iron or aluminum oxides, particularly in fersiallitic Mediterranean soil, as observed by these authors, it might be hypothesized that the clay, Fe, or Al oxides content might be predictable via fluorescence spectroscopy.

For the purpose of rapidly scanning sample series, a LED-based multiple excitation fluorescence sensor instrument [23] was used. This hand-held proxy sensor fluorometer was originally developed for in-field measurements of vegetation, in order to measure for each leaf or fruit the content of epidermal UV absorbers [24] and other leaf or fruit compounds. It was successfully used on grape berries and bunches to predict anthocyanins, flavonols and chlorophyll content at the within-vineyard scale [25]. Versions of the instrument were also used to measure blue-green autofluorescence of wheat leaves [26] and violet-blue autofluorescence of induced grapevine phytoalexins [27]. This study intends to evaluate the potential of fluorescence sensing performed on whole soil solid samples for predicting key soil properties at the scale of a Mediterranean wine estate, the varied soils of which were surveyed in a previous study [28]. Fluorescence measurements were carried out in conjunction with reflectance measurements in the Vis-NIR-SWIR range.

The aims of this work were to (i) compare the performance of prediction models from either fluorescence signals and indices or reflectance bands; (ii) evaluate the increase in model performance when coupling both types of information; and (iii) evaluate the performance of either single fluorescence signals or single fluorescence indices.

## 2. Materials and Methods

### 2.1. Study Area

The study zone is a 6 ha viticultural farm of the Vinsobres wine appellation (Côtes-du-Rhône, Southern Rhone Valley, France), the “Domaine des Chauvets” estate (44°19′06′′–44°19′44′′ N; 5°0′46′′–5°1′22′′ E, WGS, 1984). It is characterized by a diversity of soils including Red Mediterranean soils (chromic luvisols), colluvic calcisols, arenosols, fluvisols, and regosols (WRB, 2014), which develop from top to bottom of a Neogene molassic and conglomeratic plateau [28,29,30].

### 2.2. Soil Samples

A total of 146 samples originating from a field survey were collected in January 2015. The field survey consisted of 14 soil pits, from which 48 horizons were sampled, plus 98 additional soil surface samples evenly spread over the farm [28].

All soil profile horizons and soil surface samples went through physico-chemical analyses for conventional parameters [28]: particle size fractions (NF X31-107), soil organic carbon (SOC) content, calcium carbonate content (NF ISO 10693), iron content after Mehra and Jackson [31] extraction, total N (NF ISO 13878), and C/N ratio. The SOC content of these soil samples was determined by dry combustion at 900 °C according to the French norm NF ISO 10694. This technique provides the total C content, so that for calcareous samples a correction was applied from the determination of total carbonate (NF ISO 10693). The SOC content of calcareous samples was determined by subtracting carbon content from carbonates to find the net organic carbon content.

For the 48 horizons sampled in pits, analyses of pH in water (NF ISO 10390), cation exchange capacity, and exchangeable cations (Ca, Mg, Na, K, Fe, Mn, Al) by cobaltihexammine chloride dosing (NF ISO 23470) of assimilable P by the Olsen method (NF ISO 11263) were carried out.

In total, there were 19 soil properties, 10 of which described the topsoil dataset.

### 2.3. Reflectance Measurements

The reflectance spectra of the 146 air-dried and 2 mm-sieved soil samples were measured with the FieldSpec^®^three portable spectroradiometer (Analytical Spectral Devices Inc., Boulder, CO, USA) equipped with a contact probe that contains its own illumination source, a quartz-halogen bulb. The spectral range is 350–2500 nm, the spectral resolution being 3 nm in the 350–1000 nm region and 10 nm in the 1000–2500 nm region. The samples were placed in quartz dishes with dimensions of 5 cm in diameter × 0.5 cm in height. The quartz dishes were put on the vertically disposed contact probe and measurements were previously calibrated towards a round Spectralon^®^ reference panel of 9 cm diameter. Five spectra were recorded per sample. The mean reflectance spectrum was used for representing each site in calculations. The laboratory spectra were handled with a 1-nm sampling interval over the entire spectral range (2151 bands).

### 2.4. Fluorescence Measurements

Fluorescence measurements were carried out on the 146 air-dried and 2 mm-sieved soil samples with a non-contact hand-held multiple excitation fluorescence Multiplex^®^ sensor (FORCE-A, Orsay, France). Two versions of Multiplex were used: the prototype version of the Multiplex 330 with a violet-blue emission channel [27], and the Multiplex 3 (Mx3) [25] version with a blue emission channel. The signals and indices recorded by the two instruments were assigned the suffixes _flp and _mx, respectively (Table 1). The Multiplex 330 had two types of multichip LED-matrix light sources: six at 335 nm (UV) and three at 455 nm (B) from a RGB LED. It had three synchronized photodiode detectors for fluorescence recording: violet-blue 417 ± 30 nm (VBF), green 550 ± 50 nm (GF) and far red 750 ± 30 nm (FRF). The Mx3 had also three synchronized photodiode detectors for fluorescence and reflectance recording: blue 447 ± 30 nm (BGF), red 688 ± 11 nm (RF) and far red 750 ± 30 nm (FRF). It had two types of LED light sources: six at 373 nm (UV-A), and three RGB LEDs, from which only light at 516 nm (G) and 635 nm (R) was used. The blue excitation light was turned off to avoid saturation of the BGF detector. The blue emission channel of Mx3 (protected by the filter 447 ± 30 nm FWHM) yielded a BGF_UV fluorescence signal when synchronized to UV excitation, but recorded reflectance signals GR_G and RR_R that leaked through the emission filter when synchronized to G and R excitations, respectively.

Each measurement consisted of an average of 250 individual excitation flashes. In addition to the 14 original Multiplex signals available (five fluorescence signals from Multiplex 330, seven fluorescence and two reflectance signals from Mx3) (Table 1), nine available fluorescence-based indices, which were previously used in the literature for other purposes, such as simple chlorophyll fluorescence ratios, indices of phenolic maturity, and nitrogen balance indices, were also tested for predicting soil properties (Table 2).

A quantity of about 100 g of soil with ~0.3 cm layer depth was spread over a 10 cm × 25 cm black tray having a non-fluorescent coating. The sensor was laid vertically on the tray rim, and each measurement took less than one second. Three measurements were taken per sample, moving the sensor along the tray. To ensure that this sampling adequately represented the variability of the fluorescence signal, a set of 30 measurements was carried out on a limited number of samples, all along the tray.

### 2.5. Models for Soil Properties Prediction

Models for soil properties prediction relied on a series of predictive bands and signals or indices constructed from signal ratios.

From multiple bands, signals, and indices, partial least squares regression (PLSR) models [39,40] were constructed from either the reflectance spectra or the fluorescence signals and indices and combined models were constructed from both reflectance and fluorescence. Combined models relied on the Granger–Ramanathan [41] model averaging approach, which proved efficient for the determination of agronomic soil properties when using both reflectance and X-ray fluorescence spectra [42]. The Granger–Ramanathan model consists of fitting a multiple linear regression model where observed soil property values are regressed against the corresponding predictions derived from reflectance and fluorescence PLSR models (Equation (1)):

*Y = W_0_ + (W_refl_ × X_refl_) + (W_fluo_ × X_fluo_).* (1)

In Equation (1), *Y* is the vector of a measured soil property, and *X_refl_* and *X_fluo_* are the corresponding predictions from reflectance and fluorescence models, respectively. Ordinary least squares are used to solve the parameters *W*_0_ (the intercept, or a bias correction between measured values and the two models outcomes), *W_refl_* (the weight of the reflectance PLSR model), and *W_fluo_* (the weight of the fluorescence PLSR model).

From individual fluorescence signals or indices, simple linear regression (LR) models were constructed using standard ordinary least squares. A total of 21 fluorescence signals or indices were considered for PLSR, setting aside the two Mx3 reflectance signals *GR_G_mx* and *RR_R_mx,* but these reflectance signals were tested for simple LR.

All models were implemented in R version 3.2.1 [43] using the “pls” package [44] for PLSR. For each target soil property, the optimal number of latent variables was determined using the prediction residual error sum of squares (PRESS), being the lowest number of latent variables that induced a significant drop in PRESS values. As the study focused on evaluating relative improvements with different models, the leave-one-out cross-validation procedure was used [45]. For PLSR models, reflectance data were centered and fluorescence measurements were centered and variance-scaled. The quality of model fits was evaluated through the root mean squared error of cross-validation (RMSEcv), the coefficient of determination of cross-validation (*R*^2^) and the residual prediction deviation (RPD), i.e., the ratio between the standard deviation of the reference measured dataset against the RMSEcv. The RPD is widely used in soil spectroscopic studies in order to interpret the predictive ability of models [46]. According to Chang et al. [47], and Viscarra-Rossel et al. [48], larger values of RPD (≥2) indicate models with very good predictive ability (excellent when RPD ≥ 2.5); values comprised between 1.8 and 2 indicate good predictive ability; values comprised between 1.4 and 1.8 indicate models with moderate predictive ability, values between 1 and 1.4 indicate models with poor prediction ability, whereas values < 1 indicate a very poor model the use of which is not recommended.

For LR models with a single fluorescence signal or index, models were elaborated for all available ones before the model with the highest RPD was selected. A principal component analysis (PCA) was performed on the limited set of selected fluorescence signals or indices in order to identify outliers based on the Mahalonobis distance computed between PCA coordinates. The Mahalanobis threshold was set at 5% of the total sample. PLSR and LR models were compared, including or not the identified outliers.

## 3. Results

### 3.1. Description of the Dataset

Consistent with the high variability of soil types within the farm [28], the dataset is characterized by large variance for most soil properties (Table 3).

Variability is still large for the topsoil, and particularly for those soil components that are expected to fluoresce: SOC, CaCO_3_, and N_tot_. These components are correlated (very highly between N_tot_ and SOC; negatively between CaCO_3_ content and SOC); they show significant correlation coefficients with other soil properties, particularly SOC and N_tot_ with clay content and Fe content (Table 4), which increase in topsoil (Table 5).

Both fine silt and coarse sand in topsoil display a higher correlation with SOC and N_tot_ contents than for all horizons. CEC is highly correlated to CaCO_3_ and highly negatively correlated with SOC and N_tot_ contents, the opposite of pH (Table 6). Exchangeable Ca^2+^ is highly correlated with SOC and N_tot_, followed by Mg^2+^ and then K content. There is no correlation for P content with any of the potentially fluorescent components, nor for fine sand, which is not correlated to SOC and N_tot_, and is weakly negatively correlated with CaCO_3_ content.

### 3.2. Performance of PLSR Models

For 12 out of the 19 soil properties considered, PLSR models constructed from the reflectance spectra yielded RPD values higher than 1.4, predictable models, and outperformed the PLSR models constructed from the 21 fluorescence signals and indices (Table 7). As expected for leave-one-out cross-validated models, the bias was null or close to zero. Only reflectance predicted K content and Na^+^ content with fair accuracy (RPD 1.4), while it was poor with fluorescence. Reflectance-based RPD values were higher than 2 for exchangeable Ca^2+^, CEC, clay content, and SOC content and were higher than 2.5, that is, excellent, for CaCO_3_, pH, Fe and N_tot_ contents (in increasing order). Using the set of 21 fluorescence signals and indices, RPD values were lower than for reflectance-based models, but remained important for six of these properties, except for clay (1.65). Despite the lower accuracy for most properties in comparison with reflectance, only fluorescence could predict coarse silt with moderate ability (RPD 1.40), but this with only a slight improvement in RMSE compared to reflectance.

The averaged model constructed from both reflectance and fluorescence PLSR predictions brought improvement for all properties that were already correctly predicted from either reflectance or fluorescence, and particularly for those predicted with medium performance such as exchangeable Na^+^, fine silt, coarse silt, coarse sand, and fine sand (Table 8). In addition, the averaged model made fairly good predictions for properties that were previously poorly predicted, such as exchangeable Fe (named ‘Fe–cobalt’), Mg^2+^, and exchangeable K^+^. In total, model averaging enabled us to correctly predict 16 out of the 19 targeted properties, and resulted in a relative improvement of RMSE whatever the property.

It must be mentioned that, as an initial approach, simple concatenation of the reflectance spectra and the fluorescence signals and indices was attempted and tested, but was not found to be suitable. As a matter of fact, it resulted in either equal or even slightly lower performances than the reflectance spectra alone, suggesting that the specific information discovered by fluorescence signals and indices (not in wavelength units) was not accounted for.

**Figure 1:**
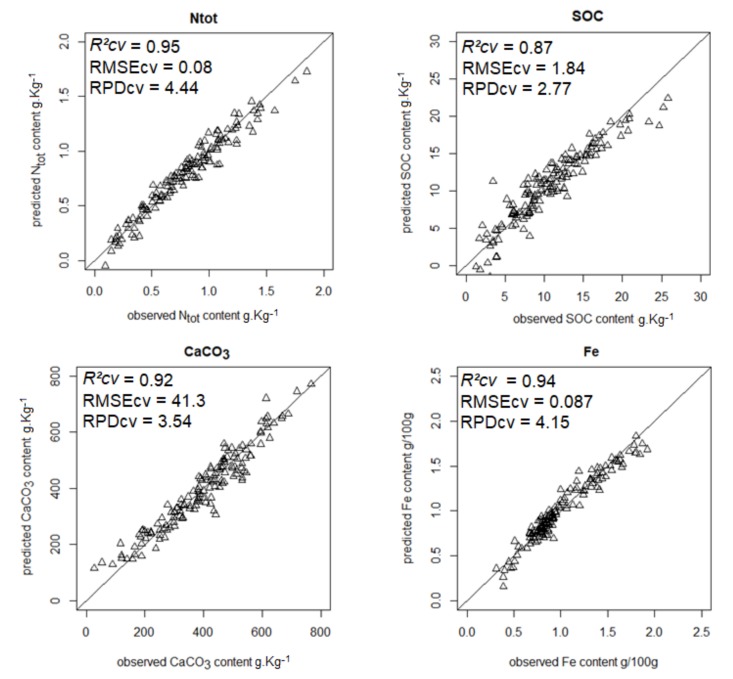
Scatterplots of predicted vs. observed Ntot, SOC, CaCO_3_, and Fe contents from model averaging.

**Figure 2:**
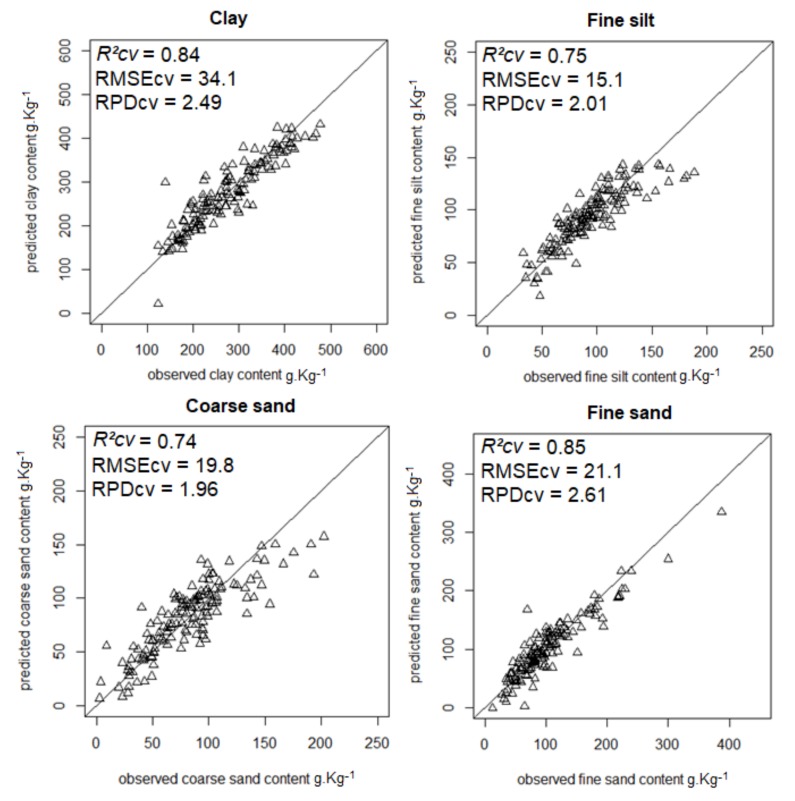
Scatterplots of predicted vs. observed clay, fine silt, coarse, and fine sand contents from model averaging.

**Figure 3:**
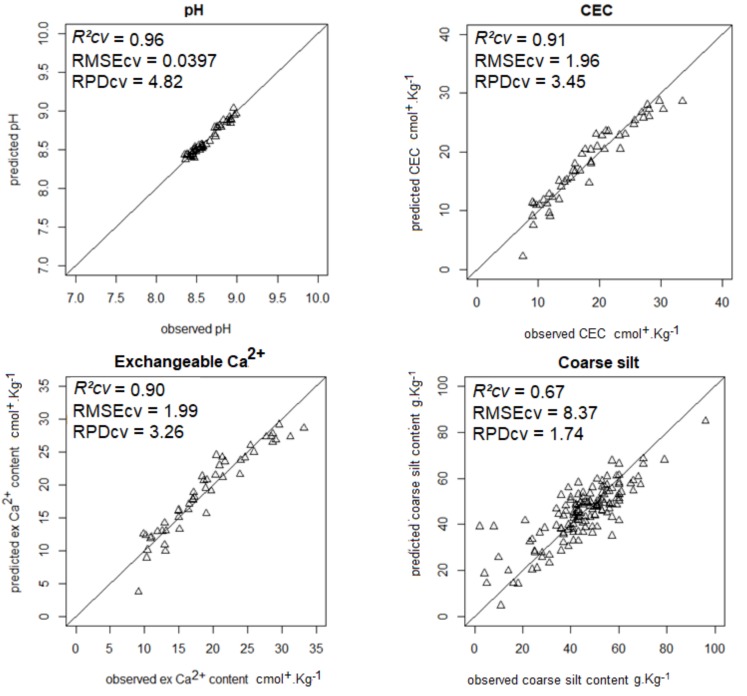
Scatterplots of predicted vs. observed pH, CEC, exchangeable Ca^2+^, and coarse silt contents from model averaging.

**Figure 4:**
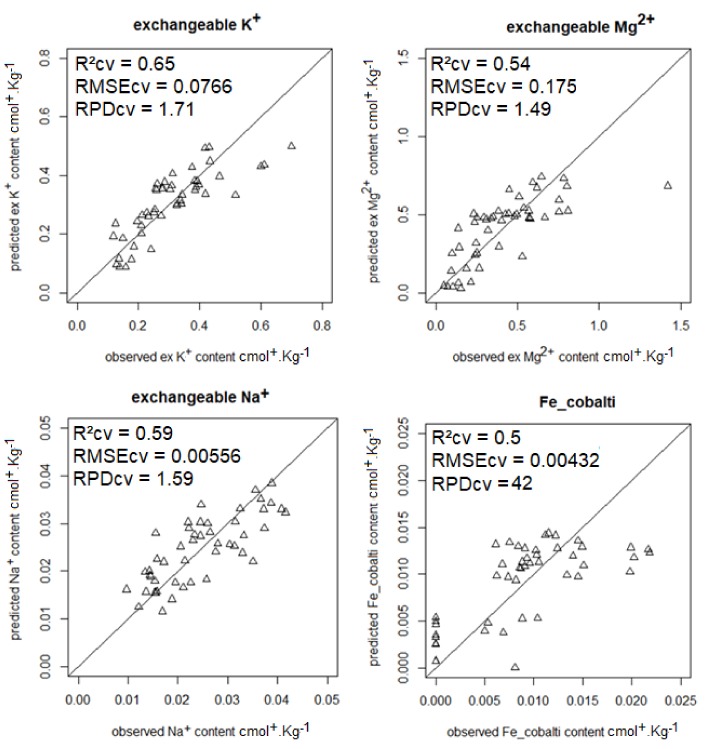
Scatterplots of predicted vs. observed exchangeable K^+^, Mg^2+^, Na^+^, and Fe (Fe_cobalti) contents from model averaging.

The most accurate predictions (RPD ≥ 1.4) are shown in Figure 1, Figure 2, Figure 3 and Figure 4.

### 3.3. Performance of Single LR Models

**Figure 5:**
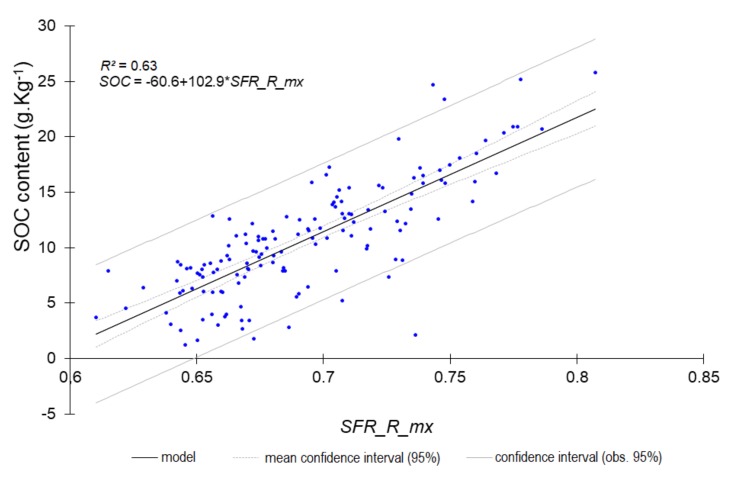
Predicted SOC content from the single *SFR_R_mx* index (all horizons).

**Figure 6:**
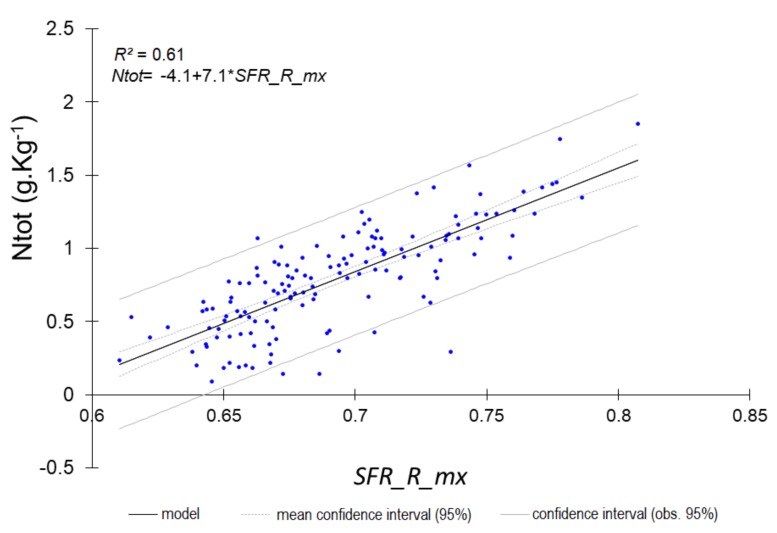
Predicted Ntot from the single *SFR_R_mx* index (all horizons).

**Figure 7:**
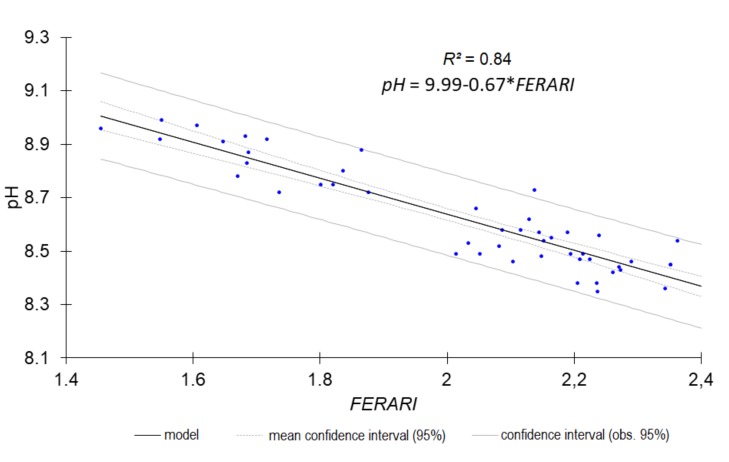
Predicted pH from single *FERARI* index (all horizons).

**Figure 8:**
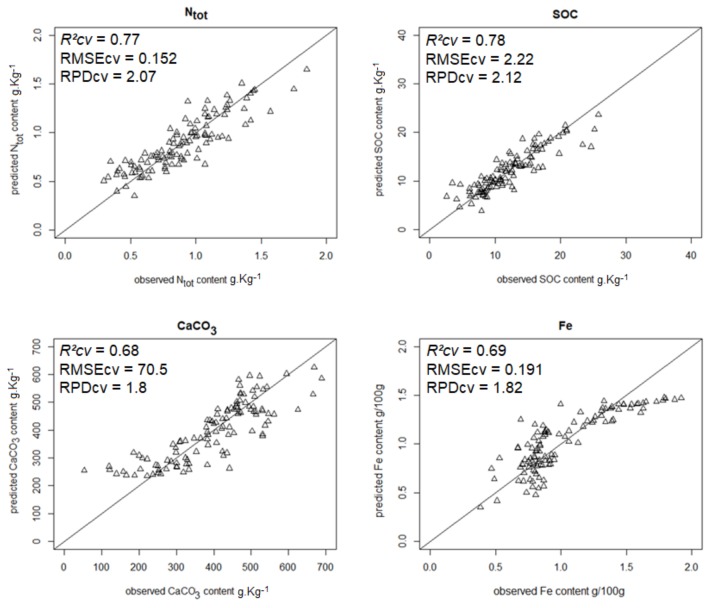
Scatterplots of predicted vs. observed topsoil N_tot,_ SOC, CaCO_3_, and Fe contents from simple linear regression models (N_tot_ and SOC: from *SFR_R_mx*; CaCO_3_ and Fe: from *RR_R_mx* and *GR_G_mx*, respectively).

The single LR models constructed from either a single fluorescence original signal or a single fluorescence index (Table 9 and Table 10, Figure 5, Figure 6, Figure 7 and Figure 8) displayed a drop in performance compared to the averaged model (Table 8). However, single LR models could predict nine soil properties out of 19 with RPD higher than 1.4, including one with excellent and one with good performance: pH (RPD 2.5) and CaCO_3_ content (RPD 1.94), respectively. Performances for clay content and exchangeable Al^3+^ content prediction were just below the 1.4 threshold (RPD 1.37). The set of best individual predictors was composed of two fluorescence indices (*FERARI_mx* and *SFR_R_mx*), one original fluorescence signal (*RF_R_mx*), and two Mx3 reflectance signals (*RR_R_mx*, *GR_G_mx*). When considering the topsoil dataset (only 10 variables described), the single LR models yielded good performance for both CaCO_3_ (RPD 1.80) and Fe (RPD 1.82) and excellent performance for both N_tot_ (RPD 2.07) and SOC (RPD 2.12) contents (Table 10, Figure 8). Topsoil coarse sand prediction yielded moderate but significant accuracy.

Except for topsoil coarse sand content, the best predictor of which stemmed from blue-excited green fluorescence, all the other best predictors were those with either red or green excitation, resulting in red or far-red fluorescence (Figure 5, Figure 6 and Figure 7), or red and green reflectance. This was rather unexpected as, according to the literature, organic matter, calcite, and quartz fluorescence are excited by the UV.

From the five main best predictors for all horizons (*FERARI_mx*, *SFR_R_mx*, *RR_R_mx*, *GR_G_m*x, and *RF_R_mx*), the outlier search based on the 5% thresholding of Mahalanobis distance led to highlighting seven outliers that appeared to be mainly the horizons of highest depth (4th to 6th horizons for 200 cm deep soil profiles), and both the highest CaCO_3_ content and lowest SOC content. However, the removal of these samples did not result in improving the prediction models; on the contrary, it lowered the performance figures. As it reduced the already limited dataset on nutrient properties (48 samples), we decided not to discard these samples.

## 4. Discussion

### 4.1. Fluorescence Is Complementary to Reflectance

In terms of performance and accuracy, reflectance spectra-based models outperformed fluorescence-based models for most properties. However, eight key properties were still well predicted from several single fluorescence signals: pH, CaCO_3_ content, Fe content, CEC, N_tot_ content, SOC content, and exchangeable Ca^2+^. Some nutrient properties such as exchangeable K^+^ and Na^+^ contents were fairly predicted from reflectance spectra only, but a particle size fraction (coarse silt) was better predicted from fluorescence, suggesting the complementarity between fluorescence and reflectance spectroscopy. Model averaging made a relative improvement in the prediction accuracy of soil properties from both reflectance and fluorescence (Table 8). An improvement between ~7% and 34% was achieved, mostly for properties with RPD ≥ 1.4, showing that there is a benefit to using both reflectance and fluorescence for the prediction of each individual property.

### 4.2. Fluorescence Single Signals May Provide a Rough Estimate in the Field

Our approach brought rather unexpected results concerning (i) the predictive ability of single fluorescence indices or original signals and (ii) the excitation wavelength that was best suited for soil property prediction. The predictive ability of single fluorescence indices or original signals was very significant for topsoil: this is important for a farmer who wishes to update information on soil nutrients for the purpose of fertility diagnosis and particularly nitrogen fertilization. These results open up encouraging perspectives, not only for using laboratory fluorescence measurements, but also for transposing this approach to the field scale, using miniaturized fluorescence devices that enable red excitation coupled with red or far-red fluorescence emissions. Miniaturization of portable hand-held fluorescence sensors is in progress. The future miniature ones, or the present Multiplex sensor (2.5 kg), which includes a GPS receiver and can be used directly in the field, would provide geospatial data and allow us to map the N_tot_ or SOC contents.

### 4.3. Red or Green Excitations Are Influential

To our knowledge, the use of green or red excitation wavelengths has rarely been attempted in previous fluorescence studies that mainly focused on UV and blue excitations for the purpose of studying humic substances. For instance, Zsolnay et al. [49], Milori et al. [13], and Tivet et al. [50] used an excitation of 440 nm, 351 nm, and 405 nm, respectively. However, Daughtry et al. [19] observed the entire excitation—emission matrix, stepping the excitation monochromator over the 250 to 800 nm wavelength range, and observed that soils had low-intensity, broad-band fluorescence emissions over the 400 to 690 nm region for excitations of 300 to 600 nm. They also noticed that soil high in CaCO_3_ content (520 g Kg^−1^) had “fluorescence intensities nearly an order of magnitude higher than the other soils observed.” Nevertheless, their objective was to discriminate between crop residues and soil, not to quantitatively assess soil properties.

The influence of blue-induced green fluorescence for predicting coarse sand topsoil content might be due to the presence of quartz, particularly in fersiallitic horizons. The reason why green or red excitations are important in predicting most other soil properties may be due to some kind of parent behavior with either chlorophyll degradation products or phenolic compounds. In particular, it may be due to cyanobacteria polypeptides such as phycobiliproteins, which characterize intracellular organic matter of dead cells and are highly fluorescent in the 550–700 nm range [51]. Cyanobacteria are present in cultivated soils but may be very scarce in Mediterranean vineyards because of higher Cu content, such as in Northeastern Italy calcaric cambisols [52]. Soil content of chlorophyll degradation products coming from plant litter is also unknown [53]. Therefore, this remains to be clarified, as along with the effects that bonding of soil constituents has on whole soil fluorescence.

### 4.4. Possible Extrapolation of These Results and Further Developments

Referring to PLSR models constructed from reflectance spectra, our results are in accordance with previous results obtained in the Vis-NIR-SWIR range overall [1,48], and for soils developed under Mediterranean or Mediterranean-like climate in particular [42,54,55,56,57,58]. Most previous assessment studies have focused on three major properties based on laboratory reflectance spectra: SOC content (e.g., [42,54,55,57,59,60]), clay content (e.g., [42,55,56,57]), and total nitrogen content (e.g., [54,57,59,60]). Other properties such as CaCO_3_ content (e.g., [57]), phosphorus content (e.g., [42,45,59]), and CEC [55,57] have been less frequently targeted and particularly exchangeable cations (e.g., [42]), such as K^+^ and Mg^2+^, which are of substantial interest for viticultural management. Our study considered a large set of soil properties with major agronomic interest, especially K^+^ and Mg^2+^ and, overall, those related to fertility management. As analyzed for spatial imagery at a regional scale [61], there are many reasons why predictions from reflectance spectra can vary in their accuracy, including: the spectral behavior of the considered property as a “chromophore” according to Ben Dor et al. [2]; the correlation of the considered property to a spectrally influent one; the internal variance of the dataset; the number and composition of sample sets; and specific soil surface conditions. PLSR loadings of spectrally influent properties such as SOC, CaCO_3_, and Fe were in accordance with their known influent wavelengths [2]. In addition to the “chromophore” behavior of properties, their specific “fluorophore” behavior is influent, either directly or indirectly. In the studied farm, Mg^2+^ and K^+^ were correlated to SOC and N_tot_ contents (Table 6), whereas P had poor correlation with every other property and gave rise to poorly predictive models (Table 7, Table 8 and Table 9). At the farm scale of three contrasted farms of Germany, Denmark, and the Czech Republic, Kuang and Mouazen [59] also found that Vis-NIR-SWIR spectroscopy was unsuccessful in predicting P content. In our study, following an eight-year shortage of chemical manure (for economic reasons), nutrient contents were very low, particularly the P content, which was even zero for about 60% of the samples [28].

Regarding the prediction of exchangeable Mg^2+^ and K^+^, it was poor or intermediate using either reflectance or fluorescence, but these two properties exhibited fair performance with model averaging. Such fair performance must be considered with caution as it is based on a rather small sample size (48 samples).

Overall, as previously achieved when combining Vis-NIR-SWIR reflectance and total X-ray fluorescence spectra [42,58,60], combining models improves the power of prediction with increased RPD and *R²* figures of merit. To our knowledge, the combining of UV-Vis fluorescence and Vis-NIR-SWIR reflectance spectroscopy has never been attempted before on solid soil samples for the purpose of soil properties assessment. For instance, Islam et al. [62] used UV-Vis-NIR-SWIR reflectance, but not fluorescence signals for this purpose.

## 5. Conclusions

Except for P content, combining Vis-NIR-SWIR reflectance spectra and a set of UV-Vis fluorescence signals enabled us to accurately predict a number of key agronomic soil properties including SOC, N_tot_, CaCO_3_, iron and particle-size contents, CEC, and pH, and to predict fairly well exchangeable K^+^, Na^+^, and Mg^2+^.

Predictions of SOC, N_tot_, CaCO_3_, iron contents, and pH were still good when using a single Multiplex signal or index such as *SFR_R*, *FERARI* or *GR_G*, or *RR_R*. The predictive ability of fluorescence indices or original signals was very significant for topsoil: this is important for a farmer who wishes to update information on soil nutrients for the purpose of fertility diagnosis and particularly nitrogen fertilization. These results open up encouraging perspectives for transposing this approach to the field: for this purpose, miniaturized fluorescence devices enabling red excitation coupled with red or far-red fluorescence emissions could be directly tested in the field.

## Figures and Tables

**Table 1:** Description of the Multiplex signals used in this study (measurement unit: mV).

Signals	Excitation Wavelength (nm)	Emission Wavelength (nm) ± Bandwidth	Sensor Version
*VBF_UV_flp*	335	417 ± 30	Multiplex 330
*GF_UV_flp*	335	550 ± 50	Multiplex 330
*FRF_UV_flp*	335	750 ± 30	Multiplex 330
*GF_B_flp*	455	550 ± 50	Multiplex 330
*FRF_B_flp*	455	750 ± 30	Multiplex 330
*BGF_UV_mx*	373	447 ± 30	Multiplex 3
*RF_UV_mx*	373	688 ± 11	Multiplex 3
*FRF_UV_mx*	373	750 ± 30	Multiplex 3
*GR_G_mx*	516	*516 leak*	Multiplex 3
*RF_G_mx*	516	688 ± 11	Multiplex 3
*FRF_G_mx*	516	750 ± 30	Multiplex 3
*RR_R_mx*	635	*635 leak*	Multiplex 3
*RF_R_mx*	635	688 ± 11	Multiplex 3
*FRF_R_mx*	635	750 ± 30	Multiplex 3

VBF, violet-blue fluorescence; GF, green fluorescence; BGF, blue-green fluorescence; GR, green reflectance; RR, red reflectance; RF, red fluorescence; FRF, far red fluorescence; UV, ultra-violet excitation; B, blue excitation; G, green excitation, R, red excitation.

**Table 2:** Description of the fluorescence indices used in this study.

Fluorescence Index	Description	Reference	Formula	Sensor Version
*BRR_flp*	Blue-to-red emission ratio	[32]	*VBF_UV_flp/FRF_UV_flp*	Multiplex 330
*SFR_G_mx*	Simple chlorophyll fluorescence ratio	[32,33]	*FRF_G_mx/RF_G_mx*	Multiplex 3
*SFR_R_mx*	Simple chlorophyll fluorescence ratio	[32,33]	*FRF_R_mx/RF_R_mx*	Multiplex 3
*FLAV_mx*	Flavonols index	[34,35]	Log*(FRF_R_mx/FRF_UV_mx)*	Multiplex 3
*FER_RG_mx*	Fluorescence Excitation Ratio	[36]	*FRF_R_mx/FRF_G_mx*	Multiplex 3
*ANTH_RG_mx*	Anthocyanins index	[37]	Log*(FRF_R_mx/FRF_G_mx)*	Multiplex 3
*NBI_G_mx*	Nitrogen Balance Index	[38]	*FRF_UV_mx/RF_G_mx*	Multiplex 3
*NBI_R_mx*	Nitrogen Balance Index	[38]	*FRF_UV_mx RF_R_mx*	Multiplex 3
*FERARI*	Anthocyanin Relative Index	[25]	Log*(1/FRF_R_mx)*	Multiplex 3

**Table 3:** Dataset used in the modeling and statistics on soil properties for either all depth horizons or topsoil horizons.

Soil Property	Description	Unit	All Horizons					Topsoil Horizons				
			Sample Size	Min	Mean	Max	sd	Sample Size	Min	Mean	Max	sd
SOC	soil organic C	g·Kg^−1^	146	1.27	10.7	25.8	5.1	112	2.6	12.1	25.8	4.73
CaCO_3_	total CaCO_3_	g·Kg^−1^	146	27	398.6	767	146.0	112	53	396	689	126.7
Iron	free iron	g/100 g	146	0.32	1.00	1.92	0.36	112	0.39	1.02	1.92	0.35
Clay	gr. fr. < 2 μm	g·Kg^−1^	146	124	274	477	84.9	112	148	280	477	78.6
Fine silt	gr. fr. 2–20 μm	g·Kg^−1^	146	33	93.6	188	30.4	112	43	93.4	188	27.5
Coarse silt	gr. fr. 20–50 μm	g·Kg^−1^	146	2	45	96	14.6	112	2	45.5	79	11.3
Fine sand	gr. fr. 50–200 μm	g·Kg^−1^	146	13	101.8	387	55.1	112	42	99	229	43.4
Coarse sand	gr. fr. 200 μm–2 mm	g·Kg^−1^	146	2	80.7	202	38.8	112	9	78.9	166	32.8
CN	C/N ratio	-	146	3.9	13.7	21.6	2.44	112	3.9	13.6	20.9	2.03
N_tot_	total nitrogen	g·Kg^−1^	146	0.09	0.79	1.85	0.35	112	0.30	0.89	1.85	0.31
pH	water pH	-	48	8.35	8.62	8.99	0.19					
CEC	cation exchange capacity	cmol^+^.Kg^−1^	48	7.44	18.1	33.5	6.8					
Ca	ex calcium	cmol^+^.Kg^−1^	48	9.11	18.9	33.2	6.48					
Mg	ex magnesium	cmol^+^.Kg^−1^	48	0.05	0.42	1.42	0.26					
Fe_cobalti	ex iron	cmol^+^.Kg^−1^	48	0	0.009	0.022	0.006					
Al	ex aluminum	cmol^+^.Kg^−1^	48	0	0.041	0.103	0.028					
Na	ex sodium	cmol^+^.Kg^−1^	48	0.010	0.024	0.042	0.009					
P	assimilable phosphorus	g·Kg^−1^	48	0	0.007	0.05	0.011					
K	ex potassium	cmol^+^.Kg^−1^	48	0.118	0.307	0.700	0.131					

gr. fr., granulometric fraction; ex, exchangeable; sd, standard deviation.

**Table 4:** Pearson correlation table of the common soil properties of all horizons (146 samples).

Variables	CN	N_tot_	SOC	CaCO_3_	Fe	Clay	Fine Silt	Coarse Silt	Fine Sand	Coarse Sand
CN	1.00									
N_tot_	−0.14	1.00								
SOC	0.12	0.96 *	1.00							
CaCO_3_	0.15	−0.55 *	−0.53 *	1.00						
Fe	−0.07	0.63 *	0.64 *	−0.87 *	1.00					
Clay	−0.06	0.63 *	0.62 *	−0.78 *	0.90 *	1.00				
Fine silt	−0.14	0.49 *	0.47 *	−0.85 *	0.78 *	0.71 *	1.00			
Coarse silt	−0.21 *	0.29 *	0.27 *	−0.68 *	0.49 *	0.38 *	0.54 *	1.00		
Fine sand	−0.21 *	−0.03	−0.07	−0.41 *	0.00	−0.15	0.19 *	0.57 *	1.00	
Coarse sand	0.06	0.24 *	0.26 *	−0.57 *	0.52 *	0.28 *	0.41 *	0.14	0.13	1.00

* Values different from 0 at a significance level of alpha = 0.05.

**Table 5:** Pearson correlation table of the topsoil properties (112 samples).

Variables	CN	N_tot_	SOC	CaCO_3_	Fe	Clay	Fine Silt	Coarse Silt	Fine Sand	Coarse Sand
CN	1.00									
N_tot_	−0.01	1.00								
SOC	0.30 *	0.94 *	1.00							
CaCO_3_	−0.15	−0.62 *	−0.65 *	1.00						
Fe	0.17	0.66 *	0.69 *	−0.89 *	1.00					
Clay	0.18	0.63 *	0.65 *	−0.80 *	0.89 *	1.00				
Fine silt	0.16	0.54 *	0.56 *	−0.87 *	0.80 *	0.75 *	1.00			
Coarse silt	0.15	0.26 *	0.31 *	−0.56 *	0.47 *	0.31 *	0.41	1.00		
Fine sand	−0.08	−0.02	−0.03	−0.22 *	−0.13	−0.34 *	0.03	0.38 *	1.00	
Coarse sand	0.07	0.39 *	0.41 *	−0.75 *	0.66 *	0.42 *	0.59	0.28 *	0.23 *	1.00

* Values different from 0 at a significance level of alpha = 0.05.

**Table 6:** Pearson correlation table of the nutrient properties of the pits’ horizons (48 samples).

Variables	CN	N_tot_	SOC	CaCO_3_	Fe	Clay	Fine Silt	Coarse Silt	Fine Sand	Coarse Sand	pH	CEC	Fe–Cobalti	Ca	Mg	Na	Al	P	K
CN	1.00																		
N_tot_	−0.37 *	1.00																	
SOC	−0.14	0.96 *	1.00																
CaCO_3_	0.51 *	−0.65 *	−0.56 *	1.00															
Fe	−0.44 *	0.75 *	0.70 *	−0.87 *	1.00														
Clay	−0.39 *	0.75 *	0.71 *	−0.78 *	0.93 *	1.00													
Fine silt	−0.53 *	0.68 *	0.58 *	−0.84 *	0.80 *	0.72 *	1.00												
Coarse silt	−0.57 *	0.39 *	0.27	−0.78 *	0.52 *	0.44 *	0.69 *	1.00											
Fine sand	−0.34 *	0.02	−0.08	−0.58 *	0.16	0.05	0.35 *	0.74 *	1.00										
Coarse sand	−0.02 *	0.22	0.22	−0.40 *	0.37 *	0.15	0.21	0.04	0.04	1.00									
pH	0.46 *	−0.87 *	−0.81 *	0.87 *	−0.82 *	−0.78 *	−0.81 *	−0.69 *	−0.37 *	−0.27	1.00								
CEC	−0.41 *	0.80 *	0.75 *	−0.83 *	0.93 *	0.96 *	0.81 *	0.50 *	0.12	0.20	−0.84 *	1.00							
Fe–cobalti	−0.30 *	0.55 *	0.53 *	−0.68 *	0.64 *	0.56 *	0.65 *	0.56 *	0.34 *	0.23	−0.70 *	0.59 *	1.00						
Ca	−0.40 *	0.79 *	0.75 *	−0.82 *	0.93 *	0.95 *	0.80 *	0.50 *	0.12	0.20	−0.84 *	1.00 *	0.61 *	1.00					
Mg	−0.31 *	0.76 *	0.73 *	−0.53 *	0.60 *	0.68 *	0.47 *	0.25	0.02	0.15	−0.71 *	0.67 *	0.51 *	0.66 *	1.00				
Na	−0.19 *	0.35 *	0.32 *	−0.57 *	0.68 *	0.70 *	0.61 *	0.37 *	0.05	0.09	−0.47 *	0.77 *	0.34 *	0.77 *	0.21	1.00			
Al	−0.25 *	0.55 *	0.54 *	−0.63 *	0.60 *	0.53 *	0.61 *	0.52 *	0.29 *	0.21	−0.68 *	0.56 *	0.99 *	0.58 *	0.53 *	0.29 *	1.00		
P	−0.04	0.26	0.27	0.02	0.02	0.01	−0.07	−0.02	−0.06	0.03	−0.16	−0.06	0.02	−0.08	0.32 *	−0.39 *	0.04	1.00	
K	−0.43 *	0.68 *	0.60 *	−0.48 *	0.41 *	0.43 *	0.46 *	0.43 *	0.21	0.09	−0.68 *	0.40 *	0.40 *	0.38 *	0.68 *	−0.12	0.40 *	0.65 *	1.00

* Values different from 0 at a significance level of alpha = 0.05.

**Table 7:** Cross-validation performance statistics of PLSR algorithm for soil properties prediction (NL_v_, number of latent variables) from either laboratory reflectance (left) or Multiplex fluorescence (right) for all horizons.

Soil Property	Reflectance (2151 Bands)				Multiplex (21 Signals & Indices)			
	*R*²cv	RMSEcv	RPD	NL_V_	*R²*cv	RMSEcv	RPD	NL_V_
CN	−0.05	2.5	0.98	9	0.01	2.42	1.01	1
N_tot_	0.92	0.099	3.57	13	0.76	0.174	2.03	4
SOC	0.81	2.21	2.31	9	0.72	2.68	1.90	7
CaCO_3_	0.88	50.0	2.92	6	0.84	58.7	2.49	7
Iron	0.90	0.114	3.17	12	0.82	0.153	2.36	7
Clay	0.78	40.0	2.12	7	0.63	51.4	1.65	8
Fine silt	0.69	16.8	1.81	9	0.49	21.7	1.40	4
Coarse silt	0.47	10.6	1.38	11	0.49	10.4	1.40	10
Fine sand	0.66	31.9	1.73	13	0.54	37.4	1.47	14
Coarse sand	0.53	26.6	1.46	13	0.41	29.7	1.31	9
pH	0.90	0.062	3.11	8	0.87	0.07	2.83	10
CEC	0.79	3.04	2.24	8	0.78	3.15	2.15	6
Fe–cobalti	0.46	0.004	1.37	2	0.47	0.005	1.38	2
ex Ca	0.78	2.98	2.18	8	0.76	3.17	2.04	3
ex Mg	0.32	0.213	1.22	3	0.32	0.21	1.23	1
ex Na	0.49	0.006	1.42	4	0.47	0.006	1.26	3
ex Al	0.42	0.0215	1.32	2	0.44	0.0211	1.35	2
P	0.12	0.011	1.08	3	-0.07	0.0117	0.97	4
ex K	0.42	0.098	1.33	8	0.32	0.107	1.22	1

ex, exchangeable.

**Table 8:** Cross-validation performance statistics of averaged model for all horizons.

Soil Property	Performance Statistics			Weights Assigned to Reflectance (*W_refl)_* and Fluorescence (*W_fluo_*) in Model Averaging		% Relative Improvement of RMSE Compared to Best Model from Either Reflectance or Fluorescence
	*R*²cv	RMSEcv	RPD	*W_refl_*	*W_fluo_*	
CN	0.21	2.17	1.13	0.96	0.14	10.3
N_tot_	0.95	0.08	4.44	0.83	0.08	19.2
SOC	0.87	1.84	2.77	0.94	0.06	16.7
CaCO_3_	0.92	41.3	3.54	0.68	0.35	17.4
Iron	0.94	0.087	4.15	0.76	0.27	23.7
Clay	0.84	34.1	2.49	0.75	0.32	14.8
Fine silt	0.75	15.1	2.01	0.97	0.04	10.1
Coarse silt	0.67	8.37	1.74	0.58	0.52	19.5
Fine sand	0.85	21.1	2.61	0.73	0.41	33.9
Coarse sand	0.74	19.8	1.96	0.76	0.35	25.6
pH	0.96	0.04	4.82	0.73	0.28	35.5
CEC	0.91	1.96	3.45	0.76	0.26	35.5
Fe–cobalt	0.50	0.0043	1.42	0.43	0.59	14.0
Ca	0.90	1.99	3.26	0.84	1.18	33.2
Mg	0.54	0.0175	1.49	0.49	0.66	17.8
Na	0.59	0.0056	1.59	0.57	0.51	6.7
Al	0.46	0.0207	1.38	0.31	0.71	1.9
P	0.14	0.0105	1.09	0.79	0.32	4.5
K	0.65	0.0766	1.71	1.06	–0.11	21.8

**Table 9:** Cross-validation performance statistics of simple linear regression from the best Multiplex signals for all horizons.

Soil Property	Single Multiplex Signal or Index			
	*R*²cv	RMSEcv	RPD	Band
CN	0.02	2.42	1.01	*VBF_UV_flp*
N_tot_	0.60	0.221	**1.60**	*SFR_R_mx*
SOC content	0.62	3.15	**1.62**	*SFR_R_mx*
CaCO_3_ content	0.73	75.3	**1.94**	***RR_R_mx***
Iron content	0.64	0.216	**1.67**	***GR_G_mx***
Clay content	0.36	62.1	1.37	***GR_G_mx***
Fine silt content	0.50	21.7	**1.40**	***RR_R_mx***
Coarse silt content	0.41	11.3	1.29	*FERARI_mx*
Fine sand content	0.15	52.1	1.06	*FRF_R_mx*
Coarse sand	0.24	34.2	1.13	*GF_B_flp*
pH	0.85	0.0766	**2.50**	*FERARI_mx*
CEC	0.64	4.22	**1.60**	***RR_R_mx***
Fe–cobalti	0.52	0.0040	**1.40**	*RF_R_mx*
Ca	0.64	4.01	**1.62**	***RR_R_mx***
Mg	0.37	0.213	1.23	***GR_G_mx***
Na	0.14	0.008	1.05	*RF_R_mx*
Al	0.49	0.0208	1.37	*RF_R_mx*
P	0.02	0.0116	0.99	*RF_R_mx*
K	0.35	0.108	1.21	*RF_G_mx*

Single signals in bold are reflectance signals.

**Table 10:** Cross-validation performance statistics of simple linear regression from the best Multiplex signals for the topsoil samples.

Soil Property	Single Fluorescence Signal or Index			
	*R*²cv	RMSEcv	RPD	Band
CN	0.12	2.12	0.96	*VBF_UV_flp*
N_tot_	0.76	0.152	**2.07**	*SFR_R_mx*
SOC content	0.78	2.22	**2.12**	*SFR_R_mx*
CaCO_3_ content	0.68	70.5	**1.80**	***RR_R_mx***
Iron content	0.71	0.191	**1.82**	***GR_G_mx***
Clay content	0.50	56.4	1.39	***GR_G_mx***
Fine silt content	0.47	20.3	1.35	***RR_R_mx***
Coarse silt content	0.24	9.98	1.13	*FERARI_mx*
Fine sand content	0.13	41.0	1.06	*FRF_R_mx*
Coarse sand	0.51	23.4	**1.40**	*GF_B_flp*

Signals in bold are reflectance signals.
